# Lineshape characterization of excitons in monolayer WS_2_ by two-dimensional electronic spectroscopy

**DOI:** 10.1039/d0na00240b

**Published:** 2020-04-21

**Authors:** Liang Guo, Chun-An Chen, Zhuquan Zhang, Daniele M. Monahan, Yi-Hsien Lee, Graham R. Fleming

**Affiliations:** Department of Chemistry, University of California Berkeley California 94720 USA grfleming@lbl.gov; Kavli Energy Nanoscience Institute at Berkeley Berkeley California 94720 USA; Mechanical and Energy Engineering, Southern University of Science and Technology Shenzhen 518055 China; Materials Sciences and Engineering, National Tsing-Hua University Hsinchu 30013 Taiwan; School of Physics and Technology, Wuhan University Wuhan 430072 China; Molecular Biophysics and Integrated Bioimaging Division, Lawrence Berkeley National Laboratory Berkeley California 94720 USA

## Abstract

The optical properties of monolayer transition metal dichalcogenides (TMDCs), an important family of two-dimensional (2D) semiconductors for optoelectronic applications, are dominated by two excitons A (XA) and B (XB) located at K/K's valleys. The lineshape of the excitons is an indicator of the interaction of the excitons with other particles and also largely determines the performance of TMDC-based optoelectronic devices. In this work, we apply 2D electronic spectroscopy (2DES), which enables separation of the intrinsic homogeneous linewidth and the extrinsic inhomogeneous linewidth, to dissect the lineshape of XA in monolayer WS_2_. With a home-built broadband optical parametric amplifier, the 2D spectra give the exciton linewidth values for extensive ranges of excitation densities and temperatures, reflecting inter-exciton and exciton–phonon interactions. Meanwhile, the time-domain evolution of the lineshape reveals a similar rate of spectral diffusion to that in quantum wells (QWs) based on III–V semiconductors.

## Abstract 2

Two-dimensional electronic spectroscopy (2DES) provides dual perspectives for characterizing exciton lineshape in monolayer transition metal dichalcogenides (TMDCs), allowing discrimination of homogeneous and inhomogeneous linewidths.

## Graphical abstract

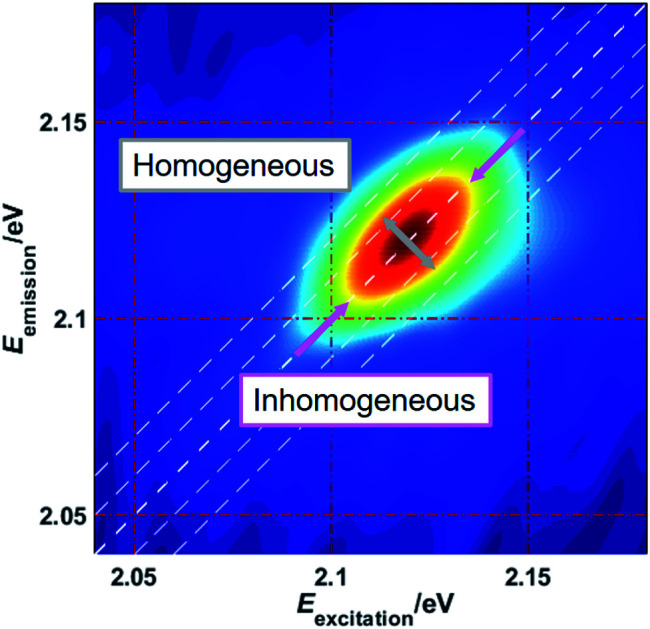

## Introduction

Monolayer transition metal dichalcogenides (TMDCs) have kindled a burst of research interest due to their salient optical properties such as direct bandgaps^1,2^ and a valley degree of freedom tunable by optical helicity.^3,4^ Quantum confinement and reduced dielectric screening in two-dimensional (2D) structures give excitons with large binding energies,^5,6^ which play a key role in the interaction of light and monolayer TMDCs. The linewidth of the excitons affects the absorption and the emission spectra, which largely determine the performance of TMDC-based optoelectronic devices. In addition, the energy fluctuation landscape is encoded in the exciton lineshape and its time-domain dynamics, which reflects the interaction of the excitons and the other particles such as phonons.^7,8^ Therefore, characterization of the exciton lineshape could provide insights for both application and fundamental research. Particularly, there has recently been a growing demand for accessing the intrinsic linewidth of the excitons in monolayer TMDCs,^9,10^ which calls for a general method for quantitatively evaluating the lineshape.

Two-dimensional electronic spectroscopy (2DES) provides a clearer method of determination of various contributions to the lineshape than absorption and photoluminescence measurements. 2DES has proved to be a powerful technique for analysing the exciton lineshape in a variety of materials such as GaAs quantum wells,^11^ CdSe quantum dots,^12^ and CdSe nanoplatelets.^13^ The amplitude of the photon echo signal is presented as a 2D function of the excitation and the emission photon energies. In such a diagram, homogeneous (intrinsic) and inhomogeneous (caused by defects, local strain, *etc.*) broadening are projected onto perpendicular directions, *i.e.*, the anti-diagonal and the diagonal directions, thus providing a readout of the two contributions.^14^ In addition, evolution of the lineshape *versus* the waiting time between excitation and emission indicates spectral diffusion caused by dynamic mechanisms such as phonon-assisted migration of excitons.^15^ During this process, the system loses memory of the excitation photon energy so that the correlation between the excitation and the emission photon energies gradually diminishes as the waiting time increases.

In particular, 2DES and also its time-domain version, photon echo spectroscopy, have been utilized to study the exciton linewidth of monolayer TMDCs.^7,16–18^ However, the laser sources used in most of the previous studies were Ti:sapphire laser oscillators, limiting the studied materials mainly to WSe_2_ and MoSe_2_ since their exciton resonance energies are covered by the laser spectrum. In ref. 17, an optical parametric oscillator (OPO) driven by an oscillator served as the laser source to access exciton A of monolayer MoS_2_. However, an OPO generally has a narrow bandwidth (long transform-limited pulse width) so that characterization of the exciton linewidth has been limited to temperatures below 40 K, above which the homogeneous linewidth becomes too wide to be well resolved (equivalently, the dephasing is too fast). A previous study applied time-domain four-wave mixing with an OPO (with a bandwidth of about 25 meV) to study the impact of the environment on exciton and trion dynamics.^19^ So far, detailed experimental analysis of the exciton lineshape with information in the frequency domain including the phenomenon of spectral diffusion for monolayer WS_2_ has been rare. As we will demonstrate later, the homogeneous and inhomogeneous widths are similar requiring accurate 2D spectra to determine these quantities by fitting to more complex functions than simple Lorentzian or Gaussian forms.

In this work, we used a Ti:sapphire laser amplifier to drive a home-built noncollinear optical parametric amplifier (NOPA), realizing broadband output covering the exciton resonance energies of monolayer WS_2_ over a wide temperature range. With this NOPA, we applied 2DES to measure the exciton linewidth of monolayer WS_2_ up to 100 K. In addition, we studied the spectral diffusion of the exciton by tracking the lineshape over the waiting time. A similar strategy could be applied for comprehensive investigation of exciton properties of the promising TMDC family, especially for MoS_2_ and WS_2_.

## Experimental methods

**Fig. 1:**
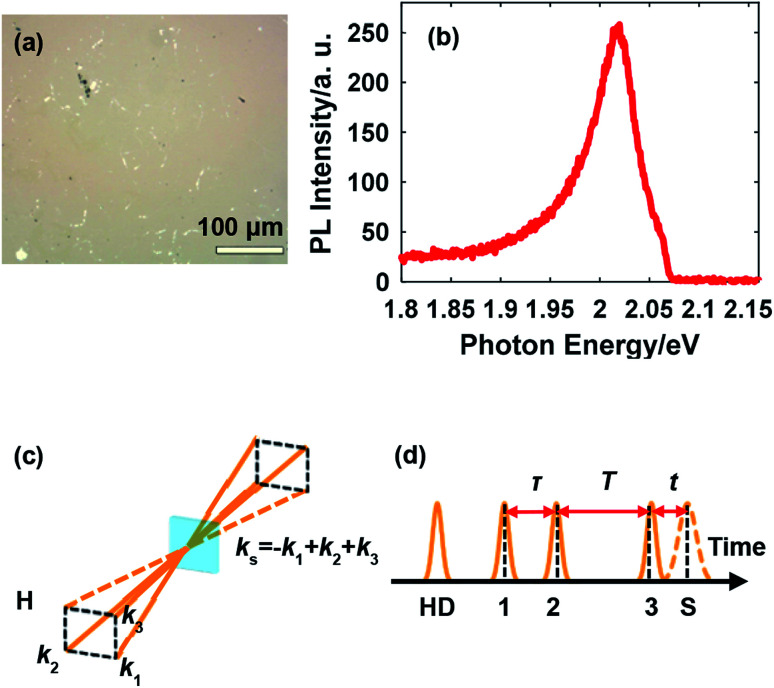
(a) Surface morphology of monolayer WS_2_; (b) PL spectrum from exciton A at room temperature; (c) the boxcar geometry for 2DES: three beams with wave vectors *k*_1_, *k*_2_, and *k*_3_ interact with the sample and the photon echo signal is emitted with the wave vector −*k*_1_ + *k*_2_ + *k*_3_; (d) the pulse sequence in 2DES for the rephasing scheme, where HD indicates the heterodyning pulse and S indicates the signal.

Continuous monolayer WS_2_ with controlled crystal quality and uniformity was synthesized on a sapphire substrate polished on one side by ambient pressure chemical vapor deposition. A quartz reactor equipped with a one-inch diameter furnace, which could precisely control the gas flow direction and adjust the reactant concentration, was utilized for synthesis. Before growth, a solution of perylene-3,4,9,10-tetracarboxylic acid tetrapotassium salt (PTAS) in deionized water was spun on the substrate. Small PTAS crystals were uniformly precipitated on the substrate after removing the water, acting as a seeding promoter. WO_3_ (99%) and S (99.5%) powders with optimized parameters were utilized for the synthesis. The growth of monolayer WS_2_ was carried out in the aforementioned furnace with a heating rate of 15 °C min^−1^ to 900 °C and a growth time of 10 min. After the growth, 100 sccm Ar flow was introduced to remove the residual reactants and the entire system was rapidly cooled down to room temperature by taking the quartz tube out of the furnace. The as-grown monolayer WS_2_ was transferred by water to another sapphire substrate polished on both sides to release the strain generated during the growth.^20^ The morphology of the sample is shown in Fig. 1(a), which illustrates the continuous distribution of monolayer WS_2_. The photoluminescence (PL) spectrum from exciton A at room temperature in Fig. 1(b) further confirms the sample quality.

A home-built NOPA was used to perform the 2DES measurements and was driven by a Ti:sapphire femtosecond laser amplifier (Astrella, Coherent Inc., 800 nm, 1 kHz). The design of the NOPA involves proper selection of the nonlinear crystal, alignment of the white light (seed) *versus* the 400 nm pump, and compensation of the pulse-front tilt between the seed and the pump.^21–23^ These considerations together enable a broadband output in the visible range fully covering the resonance energy of exciton A in monolayer WS_2_ for an extensive temperature range, which is the key for successful characterization of the exciton linewidth. The pulse was compressed using a prism pair and measured to be about 33 fs (full width at half maximum, FWHM) at the sample position. All the four beams were co-circularly polarized.

2DES was conducted in the phase-stabilized boxcar geometry involving three beams for generating the photon echo and one beam for heterodyne detection as illustrated in Fig. 1(c). The pulse sequence for rephasing 2DES, in which the photon echo is induced, is illustrated in Fig. 1(d). The photon echo signal is acquired as a function of the coherence time *τ* and the waiting time *T*. The signal is frequency-resolved in the emission photon energy domain using a spectrometer (equivalently, Fourier transformed with respect to the emission time *t*) while the resolution in the excitation photon energy domain is realized by Fourier transform with respect to *τ*, thus generating the 2D spectrum. The sample was mounted in a liquid helium cryostat for temperature-tunable measurements. A signal processing system based on two choppers and one microcontroller was described in detail in our previous work,^24,25^ and is important for ensuring the signal-to-noise ratio given the relatively low repetition rate of the laser amplifier, a tradeoff for achieving the capability of driving a broadband NOPA.

## Results and discussion

**Fig. 2:**
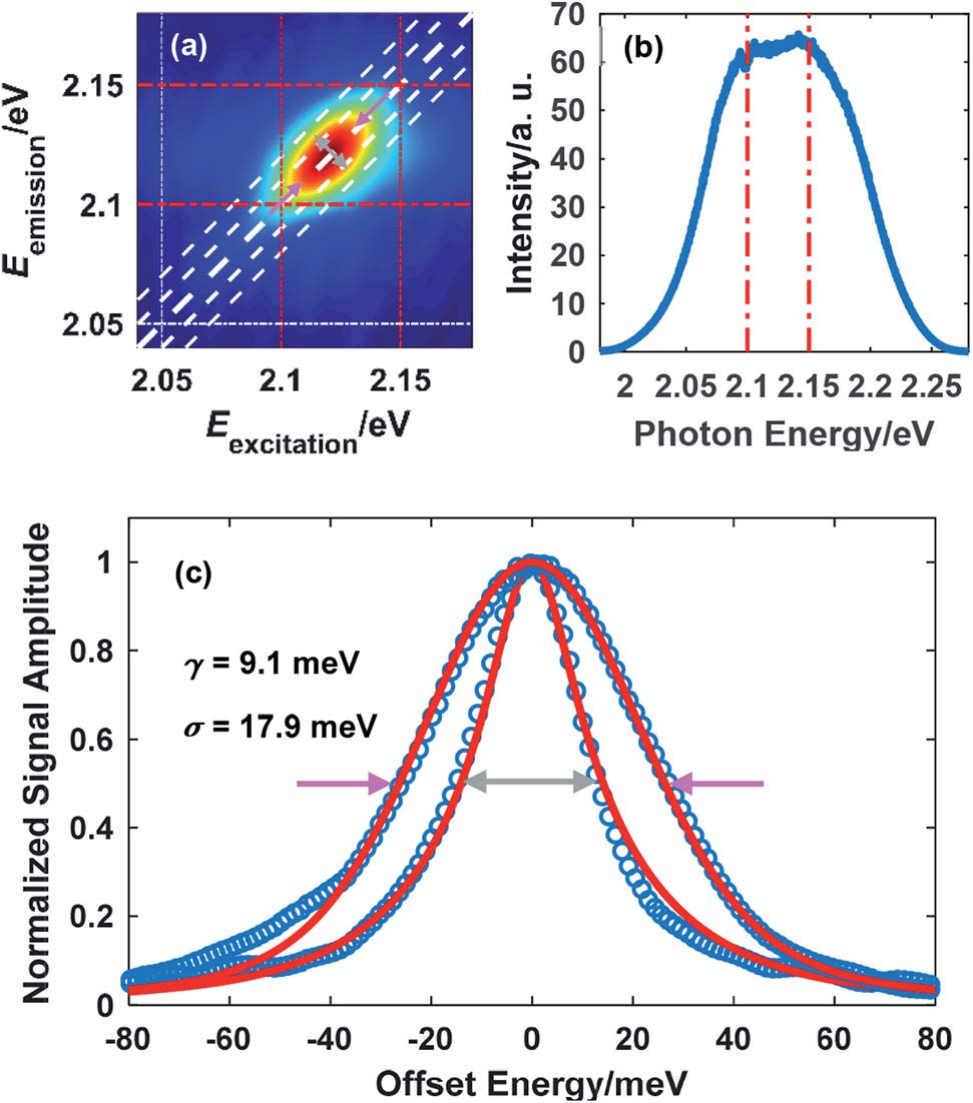
(a) A representative 2D spectrum showing the lineshape of exciton A in monolayer WS_2_ (the dot dashed lines serve as a visual guide). The temperature was 10 K and the laser fluence was 2.42 μJ cm^−2^; (b) the broadband spectrum of the NOPA output. The red dot dashed lines in (a) and (b) indicate the same spectral range: 2.1 eV to 2.15 eV; (c) extraction of the homogeneous linewidth (gray arrows) and the inhomogeneous linewidth (red arrows) by fitting using eqns (1) and (2).

A representative 2D spectrum for the lineshape of exciton A in monolayer WS_2_ is shown in Fig. 2(a). This spectrum was obtained at 10 K with a laser fluence of 2.42 μJ cm^−2^, corresponding to an exciton density of 6.79 × 10^11^ cm^−2^. The estimate of exciton density is based on the free-standing absorbance data with a local-field correction factor due to the sapphire substrate (refractive index 1.75).^26^ The waiting time is set at 100 fs unless specified. The elongation along the diagonal direction (pink arrows) indicates the inhomogeneous broadening while the width along the anti-diagonal direction (gray arrows) quantifies the homogeneous linewidth, an important intrinsic property of excitons indicative of the interaction between excitons and other particles. The laser spectrum is plotted in Fig. 1(b) with a FWHM of about 140 meV, much wider than the spectrum of the exciton resonance indicated by the red dot dashed lines. In addition, the laser spectrum near the exciton resonance is fairly flat which ensures the quality of the data on the exciton lineshape. Throughout this work, the laser spectrum was carefully tuned by adjusting the orientation of the nonlinear crystal and the delay between the seed and the pump in the NOPA to cover the exciton resonance, which redshifts as temperature increases.

From Fig. 2(a), it can be inferred that the homogeneous and the inhomogeneous linewidths have comparable magnitudes. Therefore, it is not accurate to assume that the inhomogeneous broadening dominates and that the two linewidths can be directly read. Instead, the expressions derived in ref. 14 for arbitrary inhomogeneity are applied for linewidth extraction. Briefly, the signal amplitude is related to the homogeneous linewidth *γ* and the inhomogeneous linewidth *σ* through1
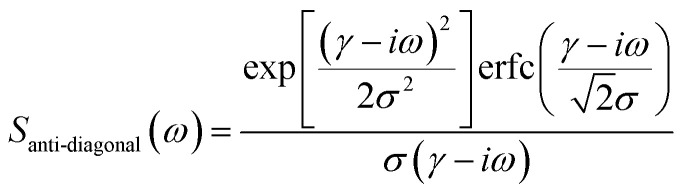
2
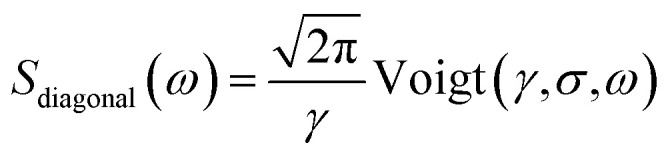
where erfc is the complementary error function and Voigt is the Voigt function, a convolution of a Lorentz function and a Gaussian function.^14^ Therefore, the measured amplitude distribution along the anti-diagonal direction and the diagonal direction can be used to determine the two linewidths. Fig. 1(c) illustrates the fitting results for the exciton lineshape in Fig. 1(a), giving *γ* = 9.1 meV and *σ* = 17.9 meV.

**Fig. 3:**
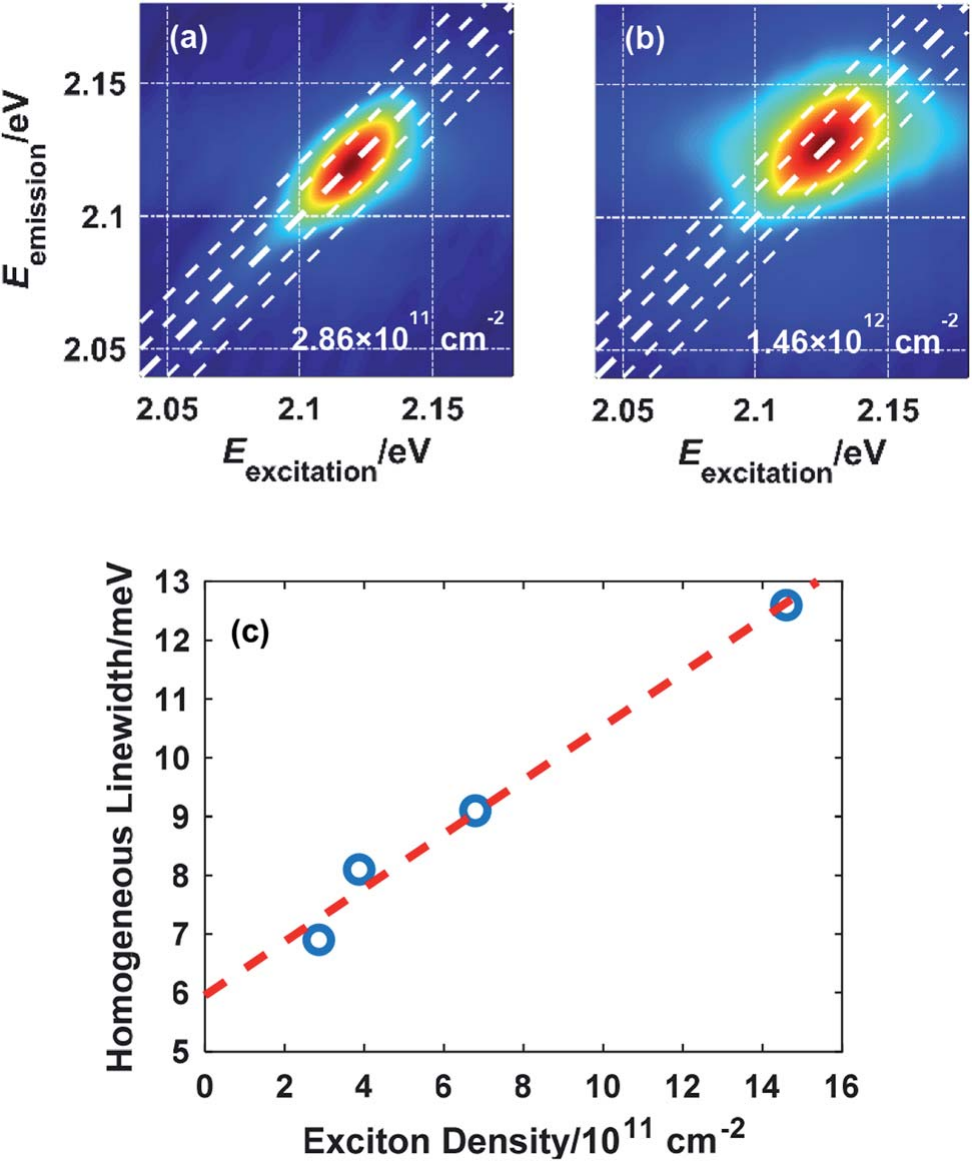
2D spectra showing the lineshape of exciton A in monolayer WS_2_ at an exciton density of (a) 2.86 × 10^11^ cm^−2^ and (b) 1.46 × 10^12^ cm^−2^; (c) variation of the homogeneous linewidth *versus* the exciton density with a linear fit to the data.

Due to the many-body interaction among excitons, the exciton linewidth is sensitive to the density of excitons. Fig. 3(a) and (b) illustrate the effect of the excited exciton density on the linewidth measured at 10 K. As more excitons are excited by increasing the laser fluence, the interaction among excitons is intensified, which accelerates the dephasing process and broadens the homogeneous linewidth, consistent with the picture of excitation-induced dephasing (EID).^7,27^ The extracted homogeneous linewidth is plotted as a function of the exciton density in Fig. 3(c). Extrapolation to zero exciton density gives a homogeneous linewidth of 5.96 meV at 10 K without external perturbation.

There is also a notable blueshift of the exciton resonance energy in the order of 10 meV when the exciton density reaches the order of 10^12^ cm^−2^, which agrees with a previous study and is due to an attraction-repulsion crossover of the inter-exciton interaction.^28^ Spectral shift of exciton resonance (either red or blue) upon excitation is universal among monolayer TMDCs resulting from this crossover mechanism, band gap renormalization, and/or exciton binding energy reduction.^28–31^ Therefore, frequency-domain analysis of the exciton lineshape is necessary to indicate whether the exciton resonance is well covered by the laser spectrum. In this work, the 140 meV FWHM of the NOPA output ensures coverage of exciton resonance.

**Fig. 4:**
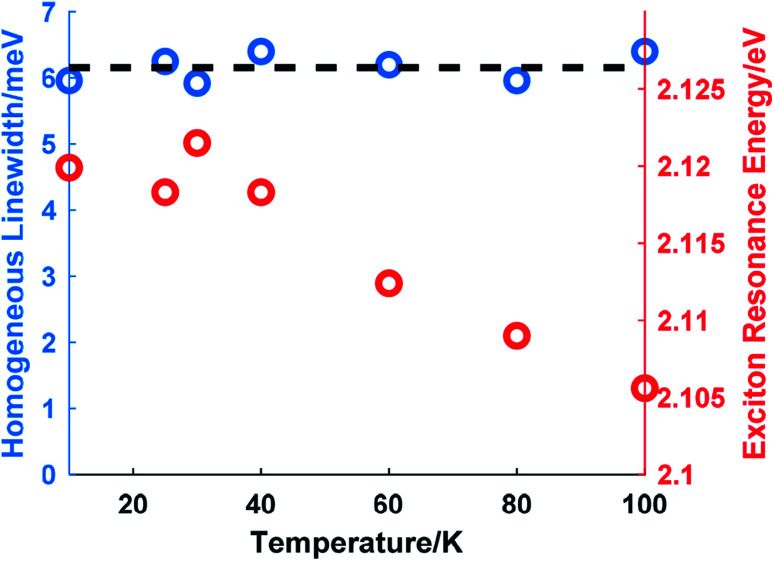
Variation of the homogeneous linewidth (left axis) and the exciton resonance energy (right axis) *versus* temperature. The black dashed line serves as a visual guide.

The temperature-dependence of the homogeneous linewidth without external perturbation was studied from 10 K to 100 K. Over this temperature range, the resonance energy of exciton A redshifted by about 14 meV as shown in Fig. 4 (right axis). The homogeneous linewidth is constant within uncertainty over this temperature range. This agrees with the experimental results in a previous study^32^ employing PL measurements and numerical deconvolution to extract the homogeneous linewidth. Since acoustic phonons dominate the phonon population at such low temperatures, the results indicate that the interaction between excitons and acoustic phonons contributes little to the homogeneous broadening and the role of optical phonons is only manifested in the linewidth above 100 K.

**Fig. 5:**
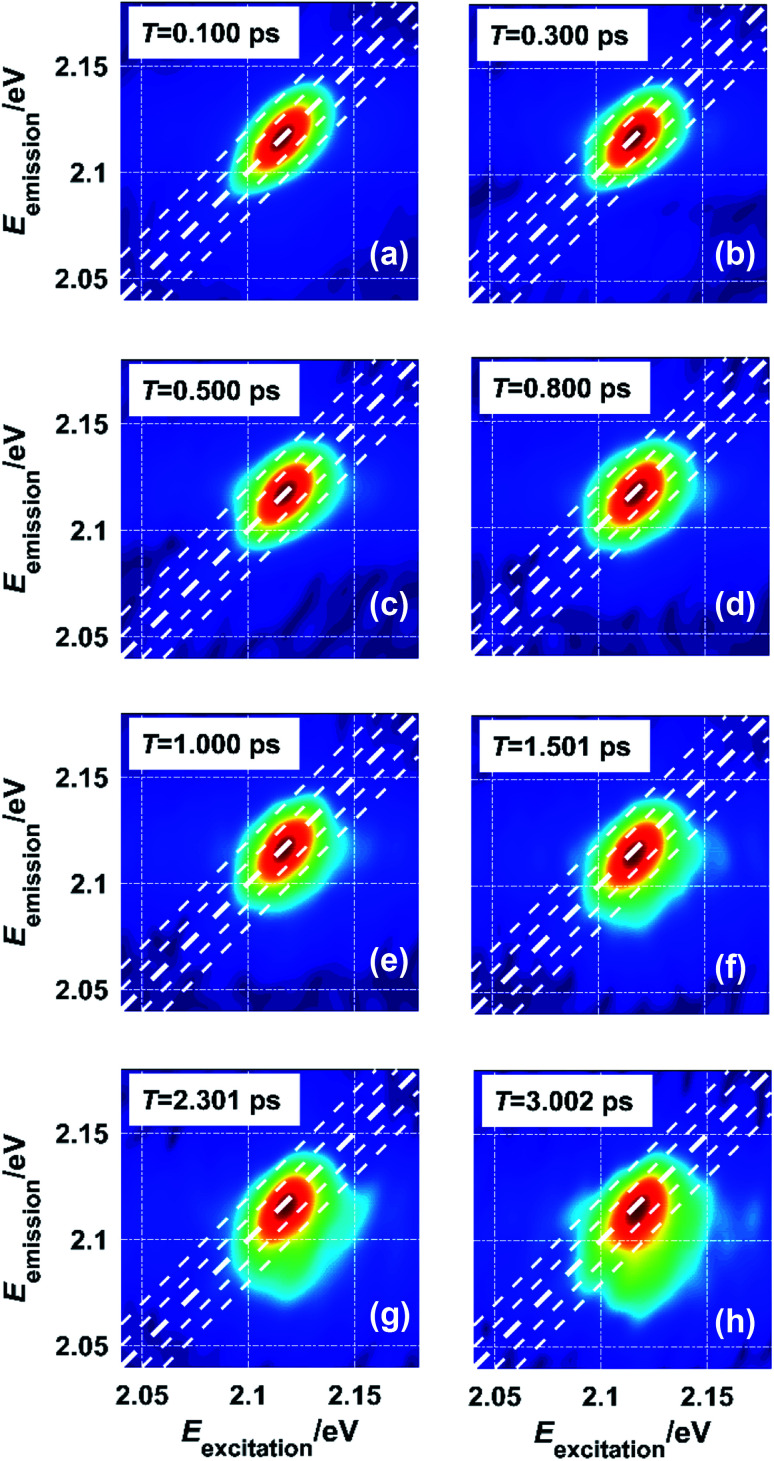
Spectral diffusion manifested in the 2D spectra by the lineshape evolution with the waiting time *T*: (a)–(h) for *T* = 0.100 ps, 0.300 ps, 0.500 ps, 0.800 ps, 1.000 ps, 1.501 ps, 2.301 ps, and 3.002 ps, respectively.

As the waiting time *T* between the excitation and the emission increases, the system loses memory of the excitation photon energy so that the correlation between excitation energy and emission energy becomes weaker. This process is reflected by the evolution of the lineshape from being elongated along the diagonal direction to being symmetric, which indicates a complete loss of the memory about the excitation. Spectral diffusion for excitons in monolayer WS_2_ was tested at 20 K with an exciton density of 6.79 × 10^11^ cm^−2^. As shown in Fig. 5, the lineshape of exciton A changes negligibly within 0.500 ps (Fig. 5(a)–(c)) and changes noticeably at later waiting times in terms of shape symmetry (Fig. 5(d)–(h)). The timescale of spectral diffusion is close to that of a GaAs quantum well at similar temperatures.^8^ However, the lineshape at longer waiting times, such as 3.00 ps, does not follow the shape predicted by free and random spectral diffusion. The intensity is concentrated more below the diagonal line than above it. This could arise from two possible causes. The first is intrinsic exciton dynamics including exciton cooling and recombination, which places more excitons in the low-energy levels. The second is the existence of traps due to defects on the sample. Such traps localize excitons and prevent spectral diffusion so that the lineshape features a combination of static disorder due to trapping and dynamic disorder due to migration. Further study is necessary to clarify the mechanism of this effect. For example, hBN-sandwiched^9,10^ or chemical-treated samples^33^ with deactivated defects could be used in control experiments to evaluate the influence of static disorder.

## Conclusions

In summary, we have applied 2DES to analyze the exciton lineshape in monolayer WS_2_ with a home-built broadband NOPA. The homogeneous linewidth of the exciton is extracted from 10 to 100 K without assumption about inhomogeneity. The homogeneous linewidth clearly rises with increasing exciton density due to the inter-exciton many-body interaction. However, the homogeneous linewidth shows no detectable change within this temperature range, indicating weak coupling between excitons and acoustic phonons. The spectral diffusion of the exciton was also characterized by following the temporal evolution of the lineshape. It is found that the lineshape evolution deviates from free spectral diffusion, possibly resulting from exciton relaxation or existence of static disorder in the sample.

## Conflicts of interest

There are no conflicts to declare.

## Supplementary Material
